# Supplementary figures and images for: Using transmission Kikuchi diffraction to characterise α variants in an α+β titanium alloy
Source: J Microsc. 2017 May 4;267(3):318–29. doi: 10.1111/jmi.12569 (PMC6849626; doi:10.1111/jmi.12569)

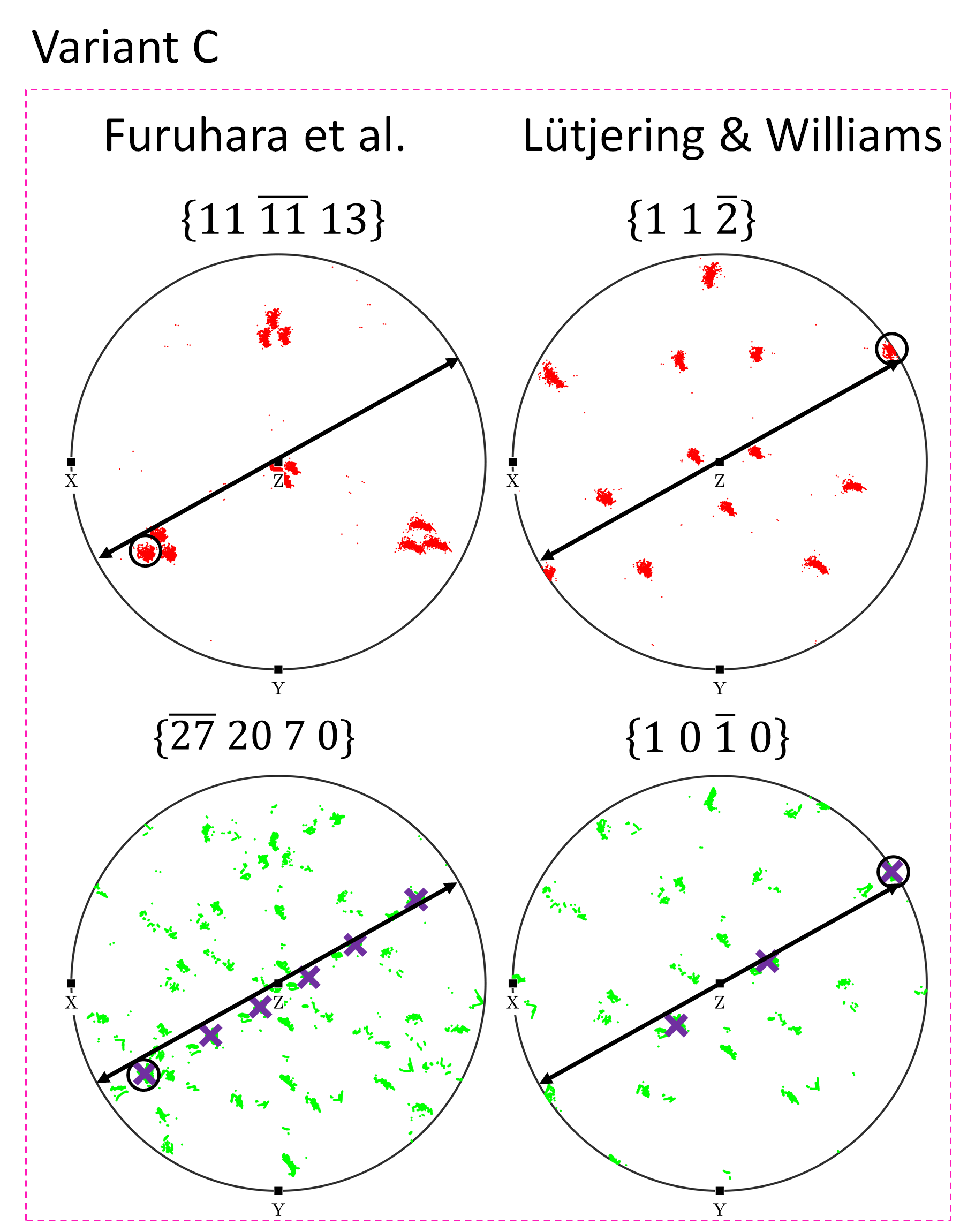

Supplement: Supplementary file 2 — Figure S1: Variant C Pole Figure Analysis. [file JMI-267-318-s002.png]

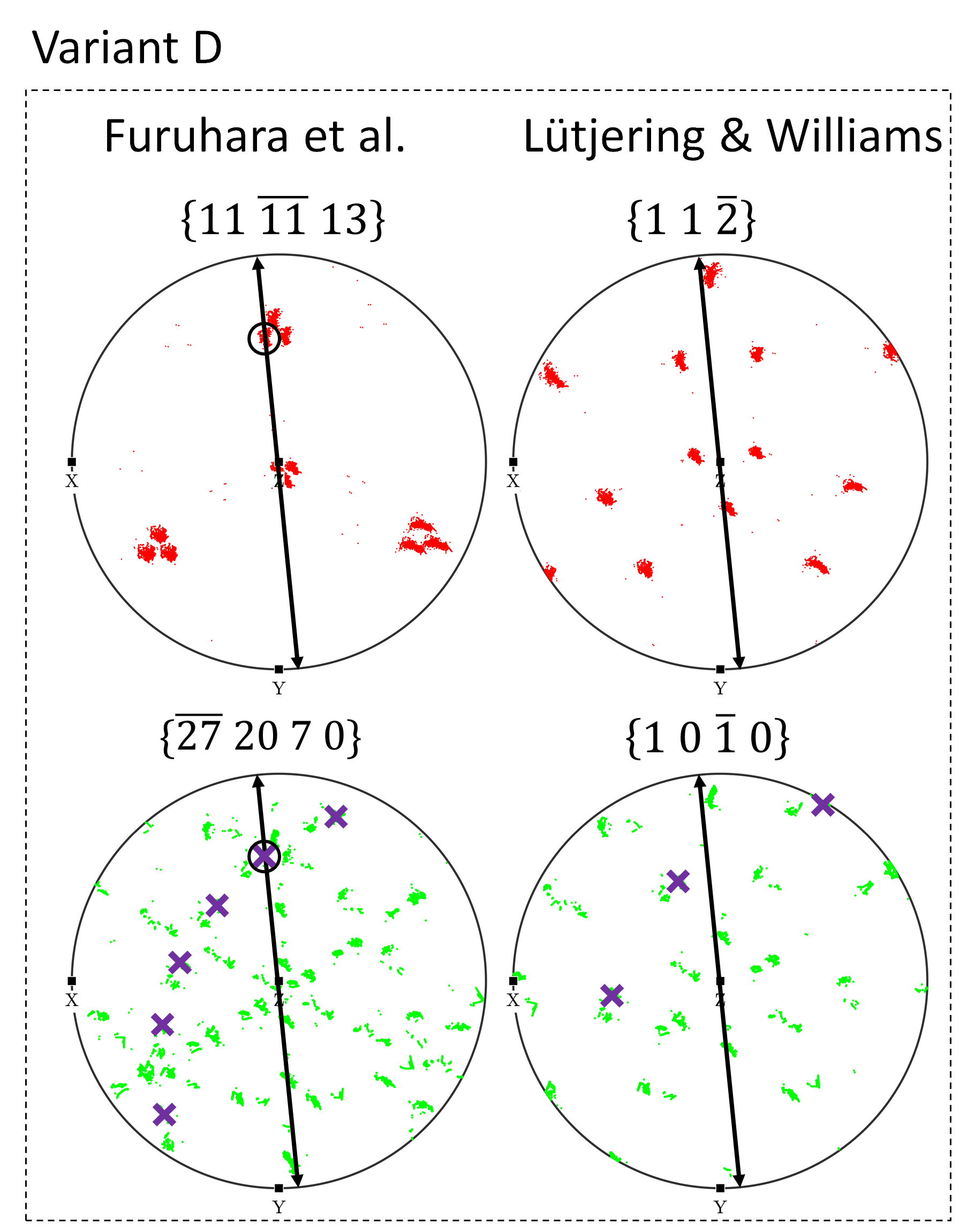

Supplement: Supplementary file 3 — Figure S2: Variant D Pole Figure Analysis. [file JMI-267-318-s003.png]

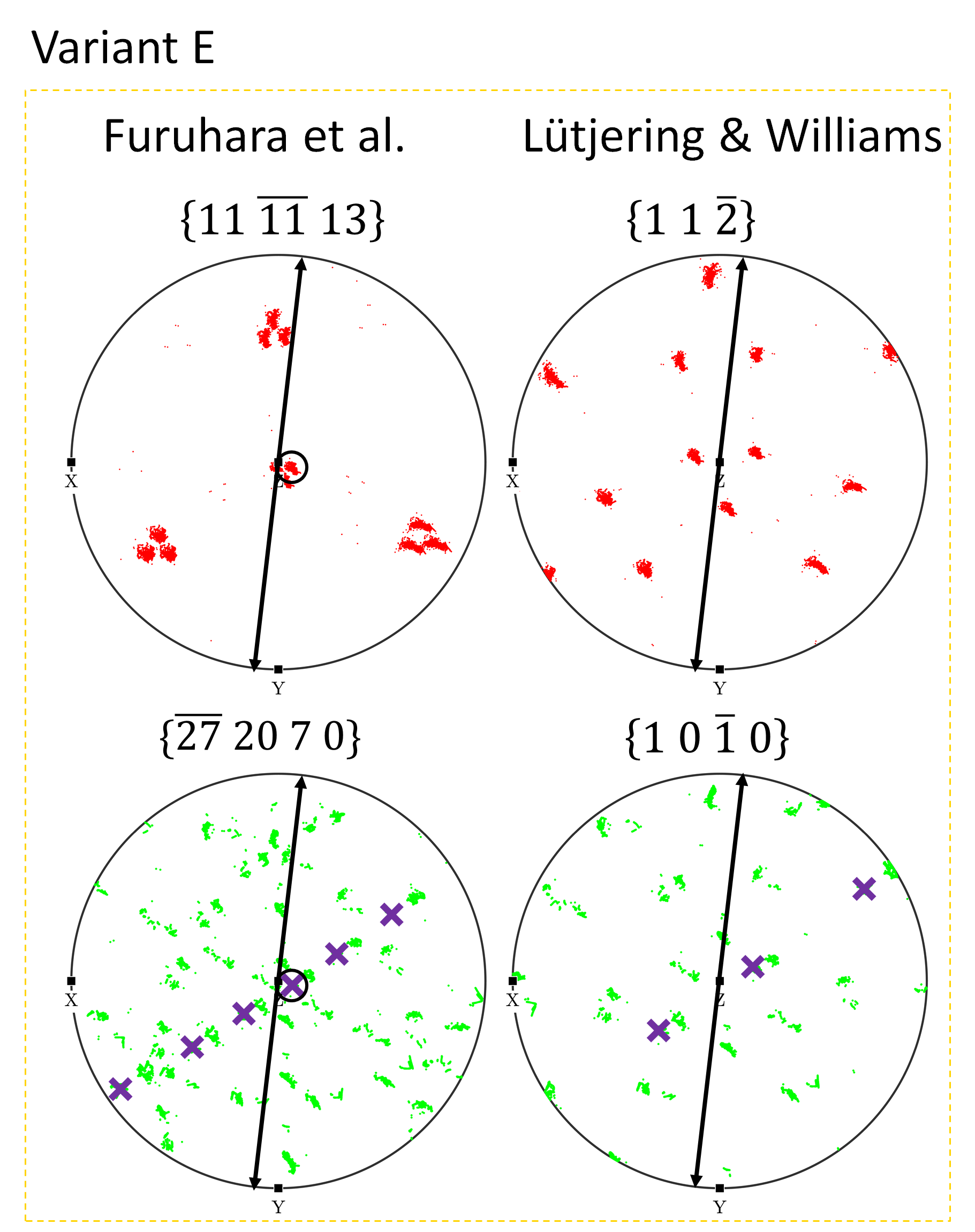

Supplement: Supplementary file 4 — Figure S3: Variant E Pole Figure Analysis. [file JMI-267-318-s004.png]

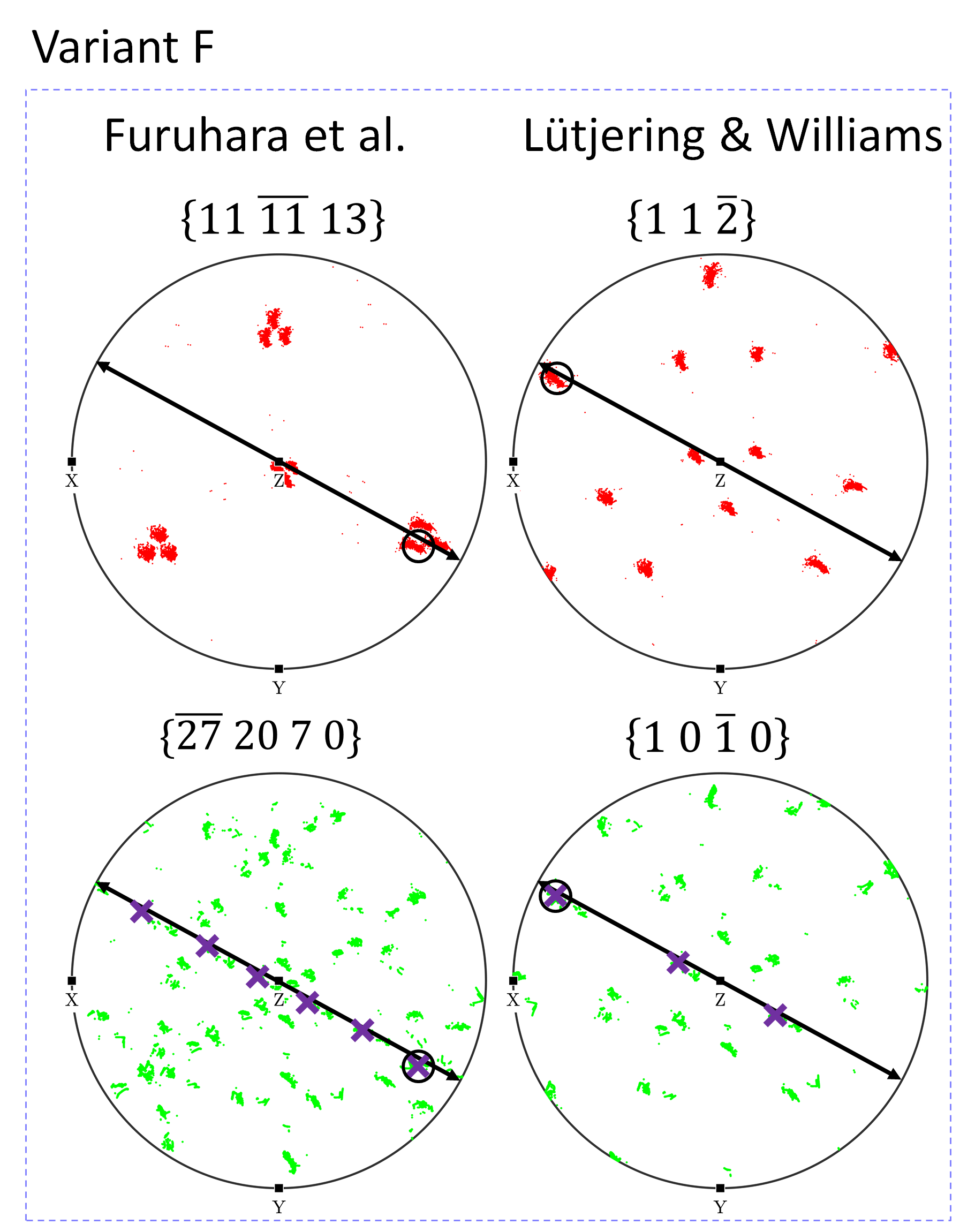

Supplement: Supplementary file 5 — Figure S4: Variant F Pole Figure Analysis. [file JMI-267-318-s005.png]

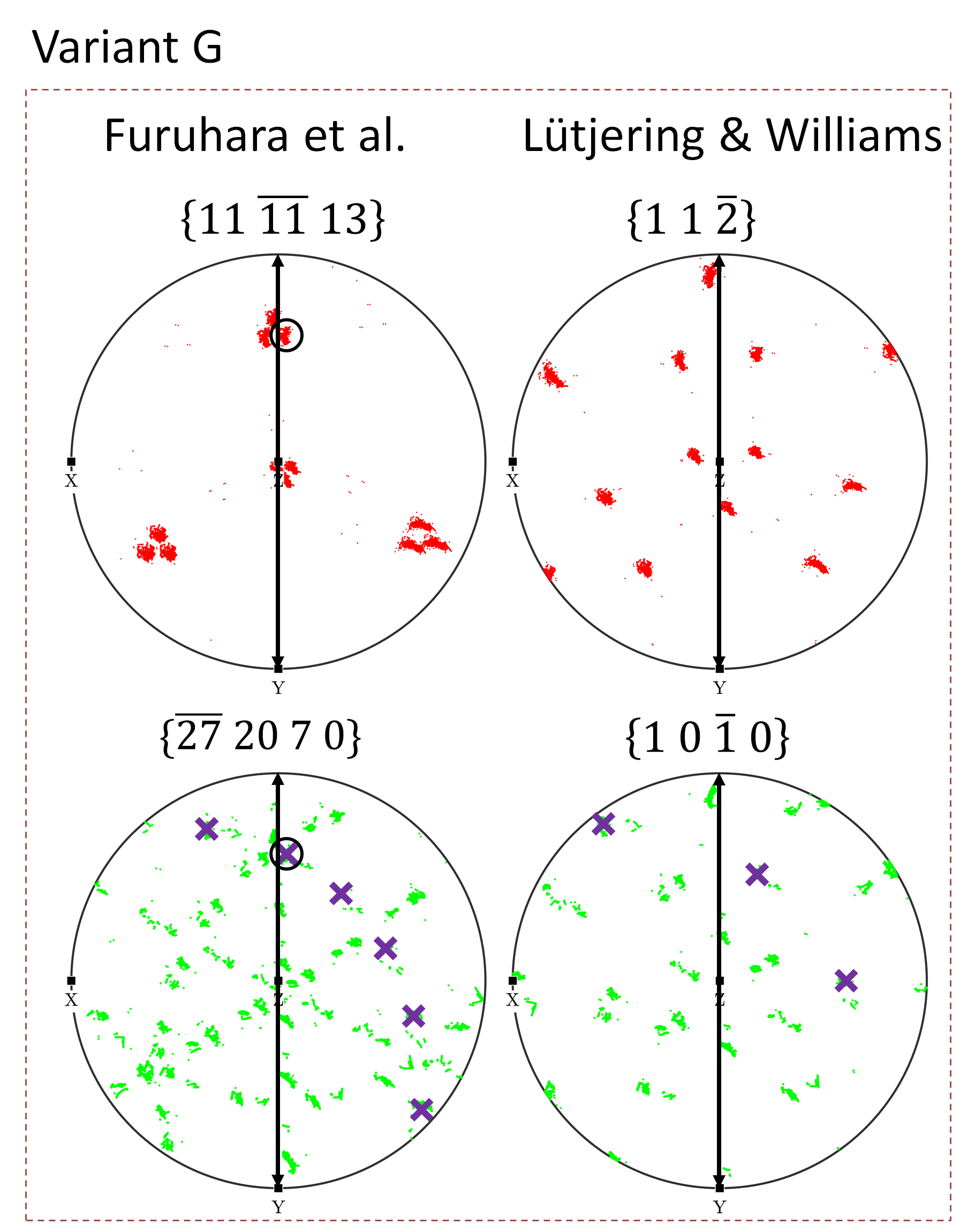

Supplement: Supplementary file 6 — Figure S5: Variant G Pole Figure Analysis. [file JMI-267-318-s006.png]

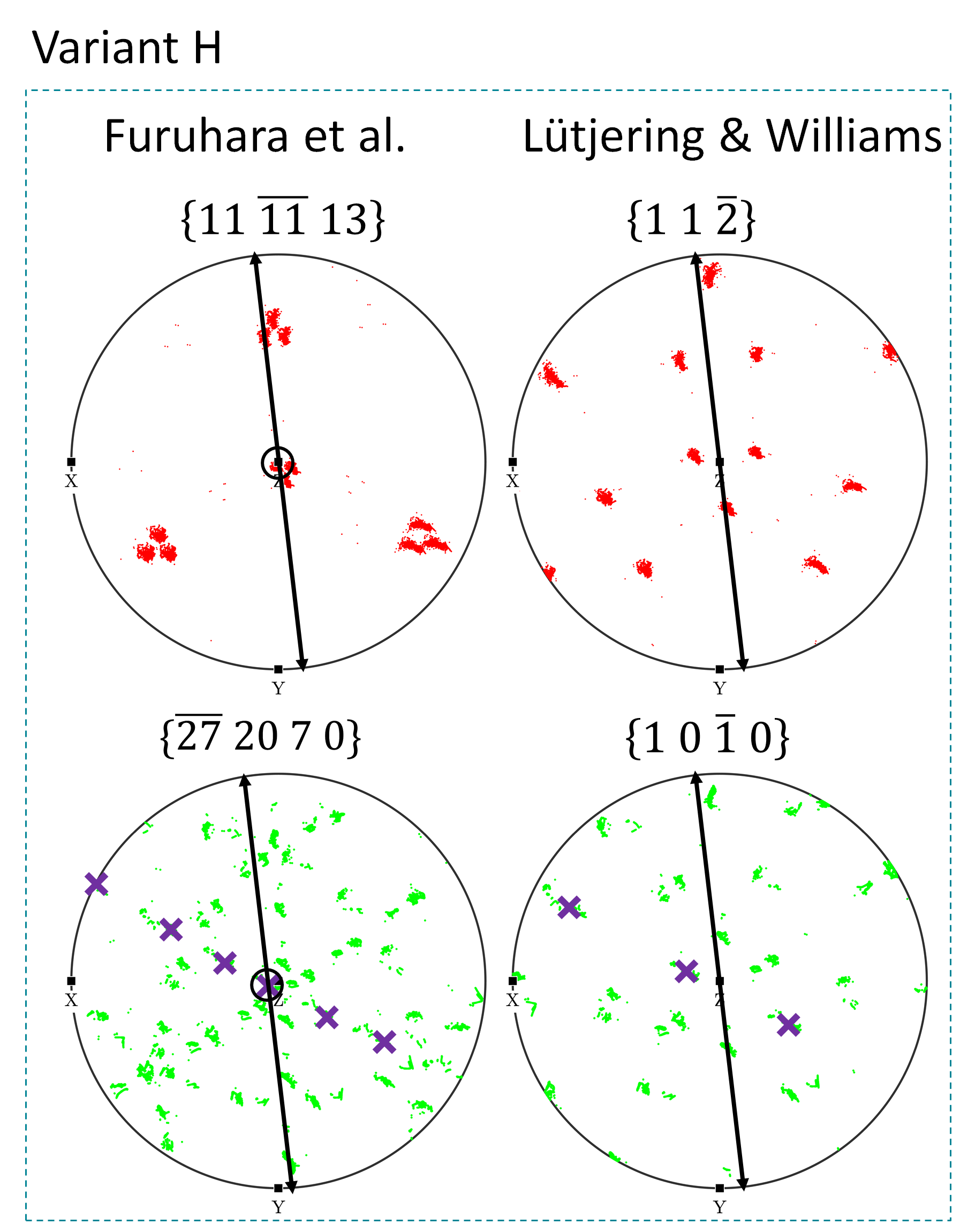

Supplement: Supplementary file 7 — Figure S6: Variant H Pole Figure Analysis. [file JMI-267-318-s007.png]

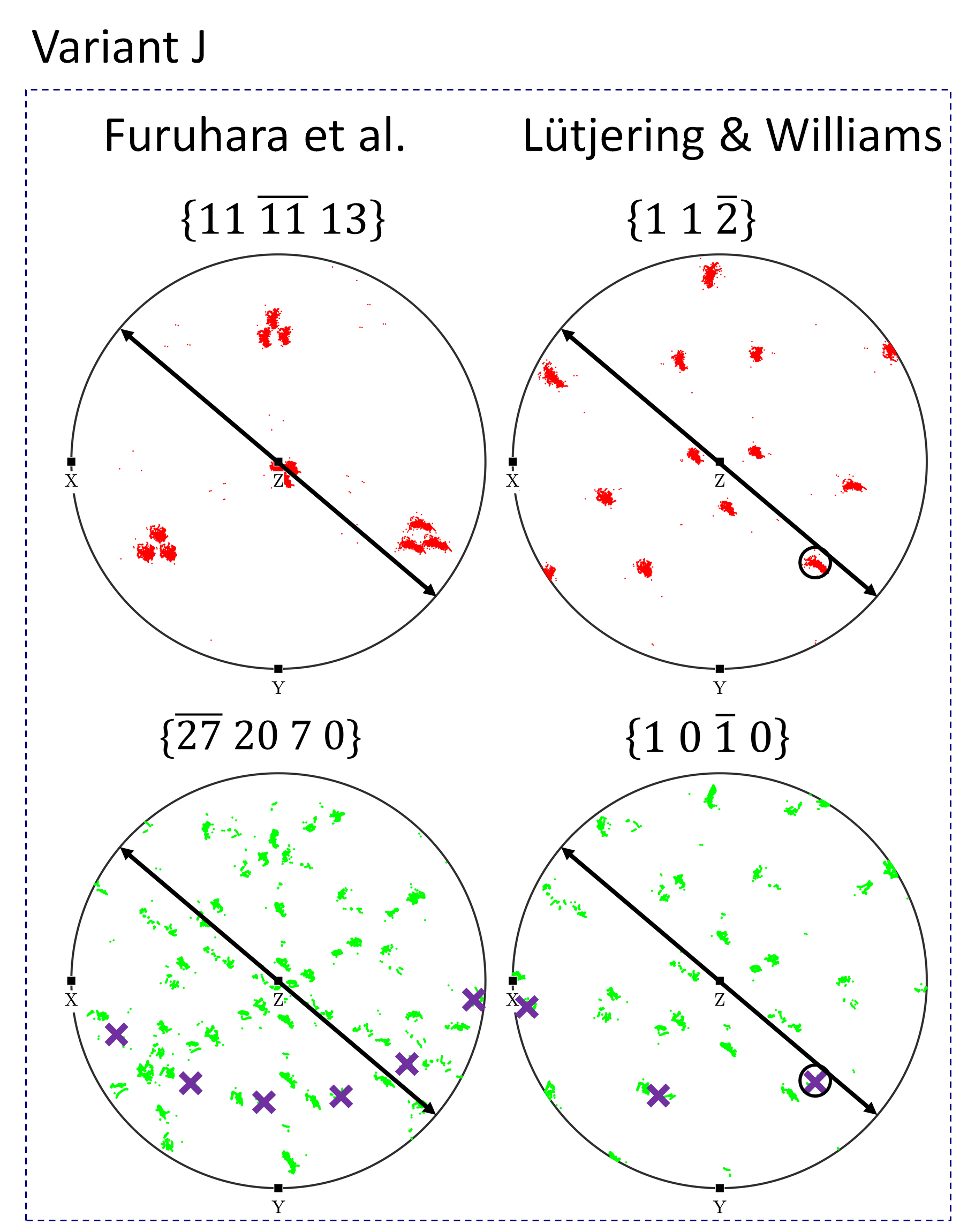

Supplement: Supplementary file 8 — Figure S7: Variant J Pole Figure Analysis. [file JMI-267-318-s008.png]

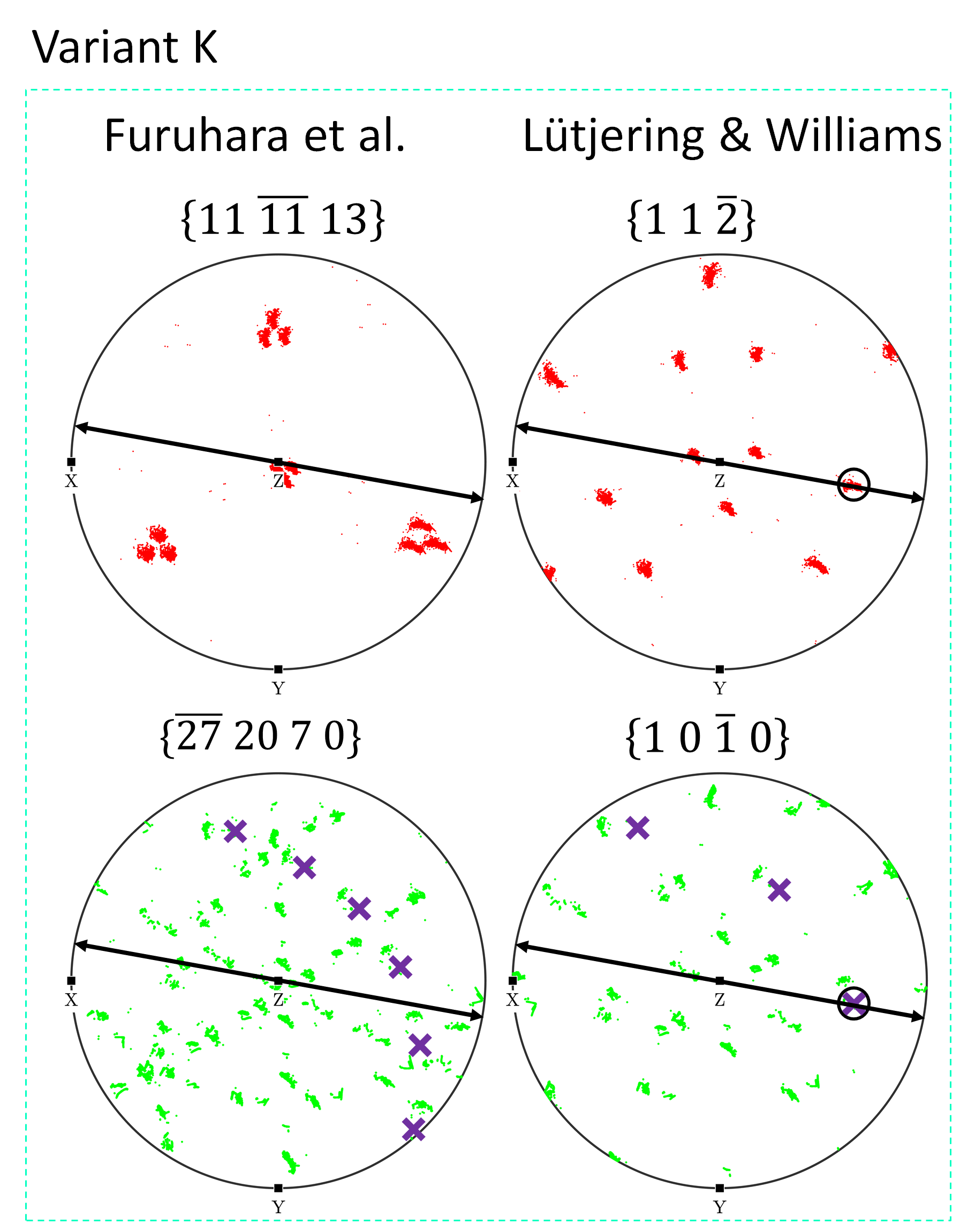

Supplement: Supplementary file 9 — Figure S8: Variant K Pole Figure Analysis. [file JMI-267-318-s009.png]

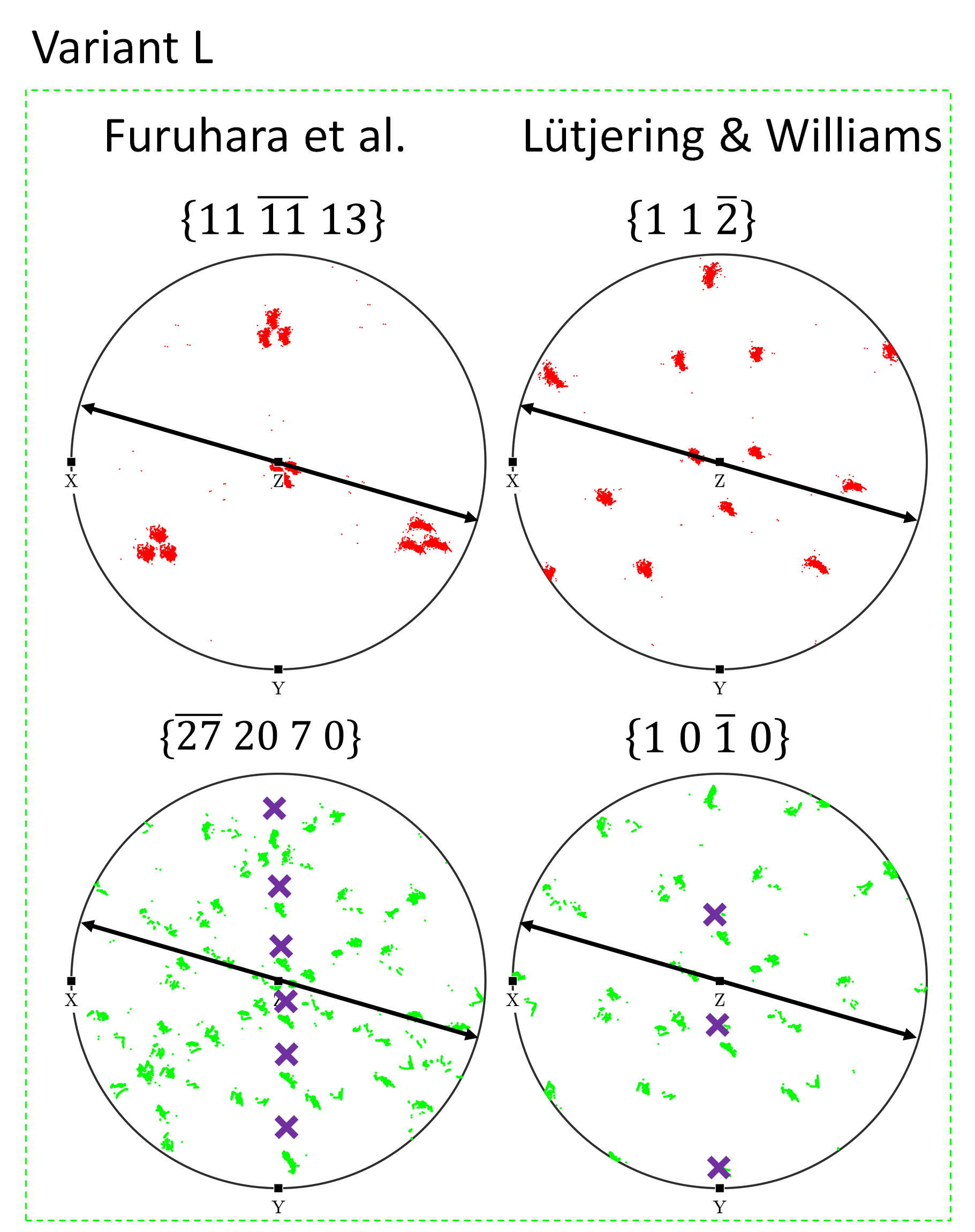

Supplement: Supplementary file 10 — Figure S9: Variant L Pole Figure Analysis. [file JMI-267-318-s010.png]

## Slide 1
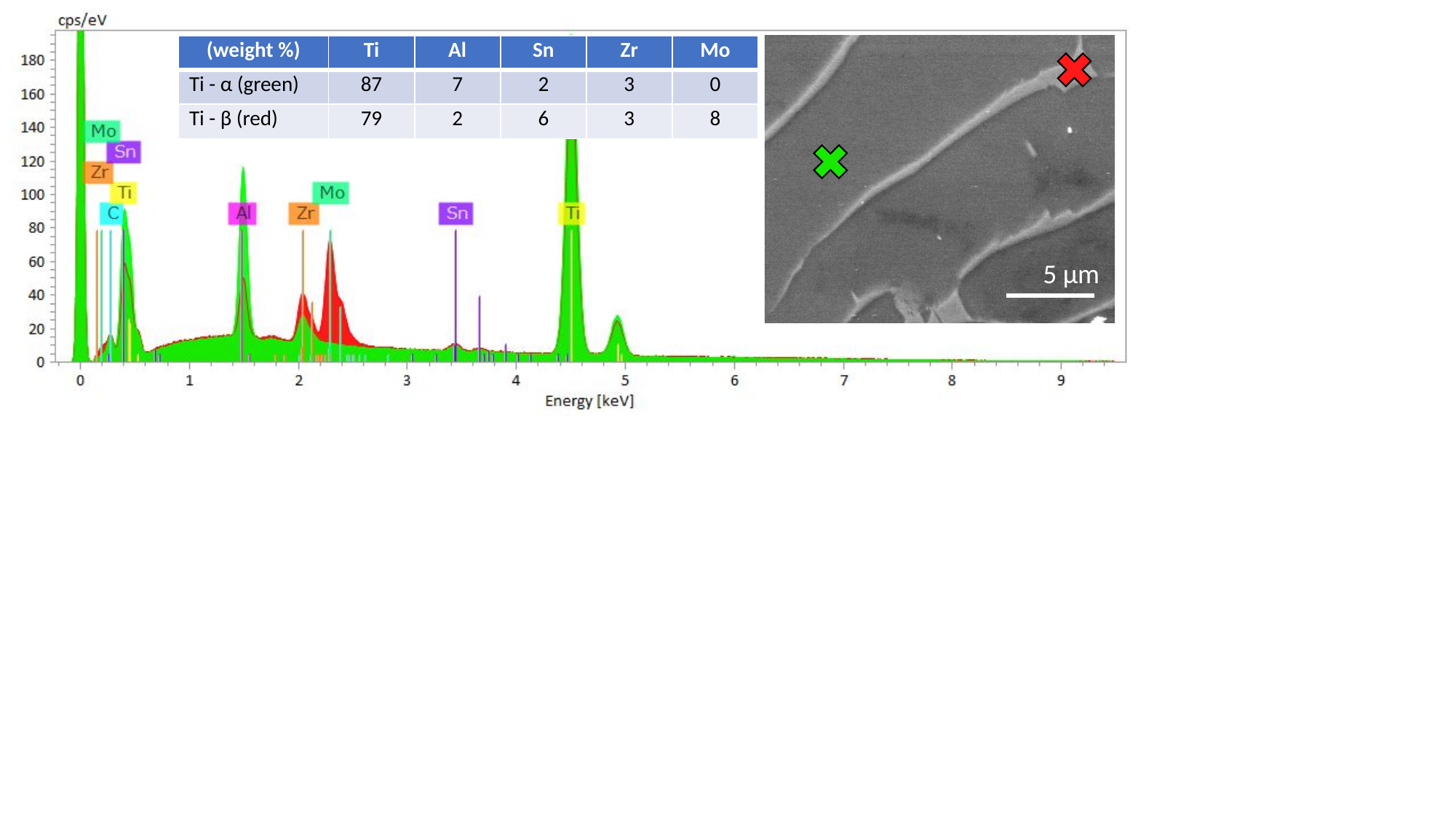

| (weight %) | Ti | Al | Sn | Zr | Mo |
| --- | --- | --- | --- | --- | --- |
| Ti - α (green) | 87 | 7 | 2 | 3 | 0 |
| Ti - β (red) | 79 | 2 | 6 | 3 | 8 |
5 µm

Supplement: Supplementary file 11 — Supplementary Data A: Supplementary Data A – EDX analysis of the alpha and beta phases. [file JMI-267-318-s011.pptx]
